# Attribute-guided prototype network for few-shot molecular property prediction

**DOI:** 10.1093/bib/bbae394

**Published:** 2024-08-12

**Authors:** Linlin Hou, Hongxin Xiang, Xiangxiang Zeng, Dongsheng Cao, Li Zeng, Bosheng Song

**Affiliations:** College of Computer Science and Electronic Engineering, Hunan University, Changsha, Hunan 410082, China; Department of AIDD, Shanghai Yuyao Biotechnology Co., Ltd., Shanghai 201109, China; College of Computer Science and Electronic Engineering, Hunan University, Changsha, Hunan 410082, China; Department of AIDD, Shanghai Yuyao Biotechnology Co., Ltd., Shanghai 201109, China; College of Computer Science and Electronic Engineering, Hunan University, Changsha, Hunan 410082, China; Xiangya School of Pharmaceutical Sciences, Central South University, Changsha, Hunan 410083, China; Department of AIDD, Shanghai Yuyao Biotechnology Co., Ltd., Shanghai 201109, China; College of Computer Science and Electronic Engineering, Hunan University, Changsha, Hunan 410082, China

**Keywords:** molecular property prediction, few-shot learning, attribute learning, meta learning, prototype network

## Abstract

The molecular property prediction (MPP) plays a crucial role in the drug discovery process, providing valuable insights for molecule evaluation and screening. Although deep learning has achieved numerous advances in this area, its success often depends on the availability of substantial labeled data. The few-shot MPP is a more challenging scenario, which aims to identify unseen property with only few available molecules. In this paper, we propose an attribute-guided prototype network (APN) to address the challenge. APN first introduces an molecular attribute extractor, which can not only extract three different types of fingerprint attributes (single fingerprint attributes, dual fingerprint attributes, triplet fingerprint attributes) by considering seven circular-based, five path-based, and two substructure-based fingerprints, but also automatically extract deep attributes from self-supervised learning methods. Furthermore, APN designs the Attribute-Guided Dual-channel Attention module to learn the relationship between the molecular graphs and attributes and refine the local and global representation of the molecules. Compared with existing works, APN leverages high-level human-defined attributes and helps the model to explicitly generalize knowledge in molecular graphs. Experiments on benchmark datasets show that APN can achieve state-of-the-art performance in most cases and demonstrate that the attributes are effective for improving few-shot MPP performance. In addition, the strong generalization ability of APN is verified by conducting experiments on data from different domains.

## Introduction

Molecular property prediction (MPP) aims to predict the physical and chemical properties, biological activity, and toxicity of molecules using computational methods, which is a fundamental step in the drug discovery process and significantly improves the efficiency of virtual screening and drug optimization [1–4]. In recent years, various machine learning and deep learning approaches have been developed for predicting molecular properties [5–8]. Mainstream MPP methods can be divided into two categories according to the way to represent molecules. The first category is sequence-based methods [9], which represent the chemical structure of a molecule as a string similar to natural language, such as simplified molecular-input line-entry system (SMILES) [10, 11], International Union of Pure and Applied Chemistry [12] and chemical identifier [13], to learn sequence-related information. Given that sequence-based methods can only extract limited one-dimensional information, the graph-based methods treat the atoms and bonds in the molecule as nodes and edges in the graph respectively to capture the two-dimensional topological structure information [14, 15].

Although these methods achieve promising performance in drug discovery tasks, they rely on the availability of extensive labeled data. However, data acquisition in this field requires expensive and inefficient cell, clinical, and other biological experiments [16–18], making it unrealistic to generate large amounts of labeled data. In response to this situation, some works combines few-shot learning (FSL) with meta-learning for MPP [19–21], including optimization-based and metric-based methods. The optimization-based methods use a model-agnostic meta-learning (MAML) strategy to optimize model initialization parameters by training on a large number of tasks, allowing it to quickly transfer to unseen tasks using only a few labeled data [22, 23]. The another several methods utilize more simpler and less costly metric-based meta-learning, such as relation network and prototype network, which learns a similarity measures through training on a large number of tasks and can quickly adapt to unseen tasks without any fine-tuning on few labeled samples [24, 25].

Currently, mainstream work focuses on extracting representations from molecular graphs, which ignores the importance of high-level concepts and makes it difficult to capture meaningful semantics for specific few-shot classification tasks. The key to success in FSL is to learn the relationship between high-level concepts and tasks [26, 27]. For example, the concept of stripes is the key to identifying zebras. In computer vision, Some recent methods utilize additional concepts such as attribute annotations or text descriptions to compensate for the lack of supervision [26, 28, 29]. By utilizing attribute information, the discriminability and generalization of the learned representations can be improved [30]. More importantly, attribute information makes the model to build a bridge between the training tasks and the testing tasks since the data in the test tasks probably contains learned attributes [30]. Unfortunately, explicitly leveraging high-level conceptual knowledge to guide model learning in few-shot property predictions has not been fully explored.

**Figure 1:**
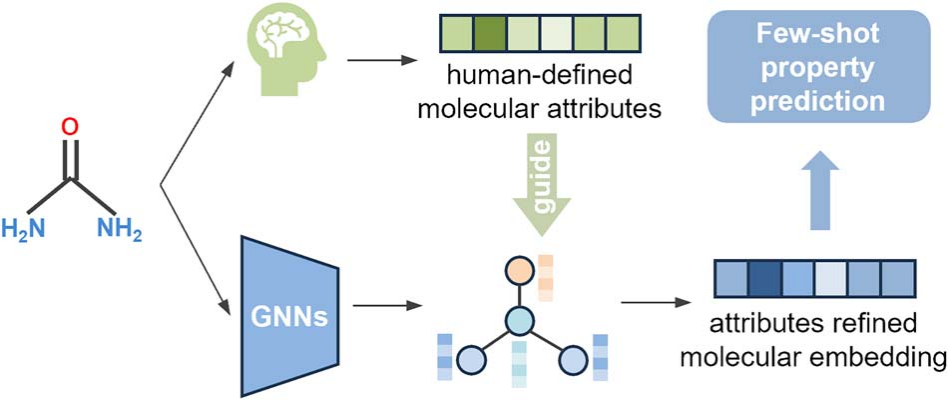
Guided by human-defined high-level molecular attribute, the molecular representation is more informative and contains more meaningful information for a specific FS-MPP task.

In this paper, we introduces a novel attribute-guided prototype network (called APN) for FS-MPP. As shown in Fig. 1, the key idea of APN is to exploit human-defined molecular attributes as high-level concepts to guide grpah-based molecular encoder for overcoming the scarcity of laboratory molecules and the insufficient generalization across different MPP tasks. Specifically, we exploit an attribute extractor, which extract molecular fingerprint attributes from 14 types of molecular fingerprints (including circular-based, path-based and substructure-based) and deep attributes from self-supervised learning methods. Then, we propose an attribute-guided dual-channel attention module (AGDA) to learn the corresponding high-level concepts in the molecular graph. In AGDA, molecular attributes are used to refine atomic-level and molecular-level representation through local and global attention, allowing APN to focus on key local and global information related to target property. Notably, the proposed APN framework is versatile and can be seamlessly integrated into any existing graph-based molecular encoder, enhancing its performance in low-data scenarios. Experimental results on multiple public datasets show the efficacy of APN in FS-MPP. Furthermore, we extensively study the impact of different molecular fingerprint attributes and deep attributes as well as combinations of different attributes on the FS-MPP. In summary, the key contributions of this work are as follows:

- We propose an APN for FS-MPP, which leverages human-defined high-level attributes to enhance the model. To the best of our knowledge, we are the first to propose the use of molecular attributes to address the FS-MPP problem.
- We design an attribute extractor that can extract molecular fingerprint attributes from 14 types of molecular fingerprints and deep attributes from self-supervised learning methods.
- We use an AGDA module, which refines atomic-level and molecular-level representations through local attention and global attention, allowing the model to learn the mapping between representations and high-level attribute concepts.
- Our experiments on multiple benchmark datasets (Tox21, SIDER, MUV, and TDC) demonstrate the effectiveness and strong generalization of the proposed framework.
- We systematically study and analyze the performance of single fingerprint attributes, double fingerprint attributes, triple fingerprint attributes, and deep attributes on FS-MPP, providing insights for future studies of molecular attributes.

**Graph-based molecular representation learning.** Molecular representation learning is a crucial area of research in computer-aided drug discovery. Its goal is to develop effective techniques for comprehending, describing, and forecasting molecular structures, properties, and interactions [31–33]. Given that molecules can be naturally represented as molecular graphs, where atoms are depicted as nodes, and bonds are depicted as edges within the graph structure, the application of graph neural networks (GNNs) and its variants [34–38] in molecular representations has gained prominence in recent years [39–41]. Graph-based molecular representation learning methods leverage GNNs to exract vector representations by capturing topological structural information inherent in molecular graphs [42, 43], which have achieved promising performance in various downstream tasks, including property prediction [44–46], drug interaction prediction [47, 48], and drug efficacy prediction [49, 50].

**Few-shot learning.** FSL aims to learn the data distribution of a task, based on only a small number of training samples [51, 52]. Currently, many tasks, such as drug discovery tasks [25] and sentiment analysis tasks [53], face the challenge of data scarcity due to the difficulty in collecting, preprocessing and labeling data, so FSL has become a promising solution [54]. In recent years, more and more FSL algorithms have combined meta-learning strategies, which learns experience or prior knowledge from a large number of tasks similar to the test tasks in the training phase, allowing it can quickly adapt to the test tasks given several labeled data [55–57]. Meta-Learning-based FSL can be classified into two approaches: optimization-based, such as MAML [58], and metric-based, such as Prototypical Network [59] and Relation Networks [60]. Optimization-based methods focus on adjusting model parameters to adapt to new tasks, whereas metric-based methods emphasize learning similarity measures for comparing examples and adapting to new tasks. In the realm of FS-MPP, the former has yielded promising results [16, 23, 58, 61], but the latter remains relatively unexplored in this domain [24, 25].

**Molecular property prediction.** MPP plays an important role in drug discovery, which is the process of using computational methods to predict molecular physical and chemical properties, biological activity, toxicity, etc. [11, 15, 32]. Considering that molecules can be naturally represented as a graph, that is, atoms are represented as nodes in the graph, and bonds are represented as edges in the graph. More and more deep learning methods are based on molecular graphs, achieving efficient and highly accurate MPP. However, due to the high demand for deep learning methods on the amount of data and the difficulty in obtaining drug data, more and more MPP methods have begun to focus on the problem of FS-MPP.

**Few-shot molecular property prediction.** In recent years, there have been some works to solve FS-MPP. An iterative refinement long short-term memory network based on matching Network is proposed [25]; Meta-MGNN combined graph neural network, self-supervised learning, and task weight aware meta-learning [61]; a property-aware embedding function are proposed to capture task-specific molecular representations, which designs an adaptive relationship graph learning module to capture the relationships between molecules [23]; and Meta-GAT use graph attention network to capture local and global information in molecules and developed a meta learning strategy based on bilevel optimization [16]. However, these methods only mine information from the molecular graph and ignore the attribute information of the molecule, which contains high-level concepts of the molecule, thereby improving the accuracy of MPP in low-data situations. APN leverage the attributes information of molecules which contain high-level molecular information defined by experts to guide deep neural networks to learn molecules. In addition, most existing works are Optimization-based, and metric-based methods have not been well discussed, which have been widely used in other fields [26, 29, 30]. APN explores the application of prototype network in the FS-MPP and proves that, with the help of molecular properties, prototype network can also achieve good performance.

## Methodology

In this section, we provide a definition of the research problem and a systematic introduction of our approach. Specifically, we first introduce the problem definition of FS-MPP (Section 5) and present the overall architecture of APN (Section 5). We then describe the details of molecular attribute extractor (Section 5) and Attributes-Guided Dual-channel Attention Module (Section 5) in APN. Finally, we elaborate the training and evaluation process of APN (Section 5).

### Problem definition

Following the setting adopted by Meta-GAT [16], we define the FS-MPP problem as a 2-way K-shot task. Therefore, the objective of APN is to utilize a set of MPP tasks, denoted as $\{T_{t}\}_{t=1}^{N_{t}}$, to train a predictor that is able to predict the labels of the molecules in new property prediction tasks given a few labeled samples. Specifically, for a new property prediction task, the $t+1$th task $\{T_{t+1}\}$ contains a support set: $S_{t+1} = \{(\mathbf{x}_{t+1,i}, y_{t+1,i})\}_{i=1}^{2K}$ whose labels is known and a query set of size q: $Q_{t+1} = \{\mathbf{x}_{t+1,j}\}_{j=1}^{q}$ for test whose labels are unknown that need to be predicted.

### Overall architecture

**Figure 2:**
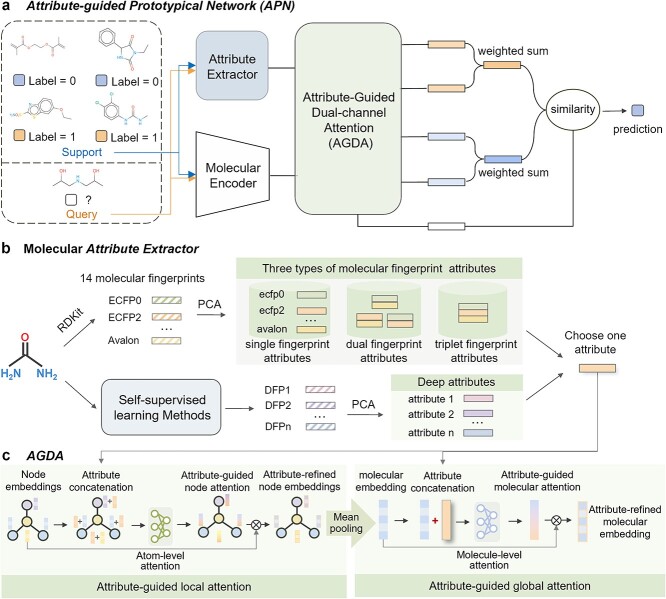
(a) The architecture of the proposed APN, where we plot a two-way 2-shot task from Tox21. APN is optimized over a set of training tasks. Within each training task $T_{t}$, the support set is used to obtain the prototypes for each class and the query set is used to optimize the parameters of the moleclue encoder and AGDA module. A query molecule $x_{t}$ is represented as $\mathbf{z}^{\prime}$ by the moleclue encoder and AGDA module, which is used to compare the similarity with prototypes for the final prediction. (b) The attribute extractor. The attribute extractor can not only extract three types of fingerprint attributes from 14 molecular fingerprints, including single fingerprint attributes, dual fingerprint attributes, and triplet fingerprint attributes, but also extract deep attributes through self-supervised learning methods, where DFP1, DFP2...DFPn are deep fingerprints generated by self-supervised learning methods 1 to $n$. (c) The overall framework of the proposed AGDA. All nodes representations of a molecule sequentially pass a attributes-guided local-attention module and a attributes-guided global-attention module to obtain the final attributes-refined molecular representation.

The overall architecture of the proposed Attributes-Guided Molecular Property Prediction (APN) is shown in Fig. 2(a), which mainly includes an attribute extractor and an AGDA module. Firstly, a molecular encoder (e.g. GAT) is used to extract representations from molecules. Then, we refine these molecular representations by taking into account the molecular attributes. Specifically, the molecular attributes generated by an attribute extractor refine the molecular representation to make it more informative and discriminative through a dual-channel attention mechanism. Finally, taking into account that each molecular representation in the support set contributes differently to the prototype, we calculate the prototypes of positive and negative examples separately in a weighted manner.

### Molecular attribute extractor

The motivation of molecular attribute extractor comes from the fact that an image can be described by several discrete and human-oriented high-level knowledge in the field of computer vision. Taking bird classification as an example, we can uniformly summarize these high-level knowledge into attribute information, including single attributes (such as feather color), combined attributes (feather color + eye color) and text descriptions (’A white bird stands on the branch’). Through this high-level semantic knowledge, we can easily generalize it to more scenarios, such as the classification of horses and tigers. Inspired by this, we find that the attributes in molecules are not fully utilized in FSL and molecular fingerprints and self-supervised learning methods can provide high-level knowledge, including chemical structure, physicochemical properties, and characteristics defined by humans. Therefore, we propose extracting molecular attributes from 14 types of molecular fingerprints (including circular-based, path-based, substructure-based, and physicochemistry-based fingerprints) and 7 state-of-the-art self-supervised learning methods, including sequence-based (molformer [62]), graph-based (CGIP [46], GraphMVP [63], MoleBERT [64], unimol [33]), and image-based models (IEM [65], VideoMol [66]). Table 1 provides detailed definitions of 14 types of fingerprints. For more information of these fingerprints, see Appendix B.

**Table 1:** The detailed information of 14 kinds of fingerprint.

No.	Type of fingerprint	Name	Dimension
1	Circular-based	ECFP0	1024
2		ECFP2	1024
3		ECFP4	1024
4		ECFP6	1024
5		FCFP2	1024
6		FCFP4	1024
7		FCFP6	1024
8	Path-based	RDK5	1024
9		RDK6	1024
10		RDK7	1024
11		HashAP	1024
12		HashTT	1024
13	Substructure-based	MACCS	167
14		Avalon	1024

Figure 2(b) shows the pipeline of the molecular attribute extractor. For the fingerprint attributes, we first use the RDKit library [67] to generate 14 types of fingerprints $\mathcal{Q}=\left \{ \bigcup _{l=1}^{14} \mathcal{Q}^{l} \right \}$ for all $n$ molecules, where $\mathcal{Q}^{l}\in \left \{ \bigcup _{i=1}^{n} q^{l}_{i} \right \}$ and $q^{l}_{i}$ represents the $l$th fingerprint of the $i$th molecule. Since most molecular fingerprints have high dimensions, we employ PCA (Principal component analysis) technology $\phi $ [68] to reduce the dimensionality down to 100 dimensions, denoted as $\mathcal{C}=\phi (\mathcal{Q})$ and $c_{i}^{l}\in \mathbb{R}^{100}$. Then, we use $\mathcal{C}$ to generate three types of fingerprint attributes $\mathcal{A}^{1}=\mathcal{C}$, $\mathcal{A}^{2}=\left \{agg(c_{i}^{k},c_{i}^{l}) | c_{i}^{k} \in \mathcal{C}, c_{i}^{l} \in \mathcal{C}\right \}$, and $\mathcal{A}^{3}=\left \{agg(c_{i}^{k},c_{i}^{l},,c_{i}^{m}) | c_{i}^{k} \in \mathcal{C}, c_{i}^{l} \in \mathcal{C}, c_{i}^{m} \in \mathcal{C}\right \}$, where $agg(\cdot )$ represents aggregation function, such as concatenating or summing. In the following, we use the lowercase form of the fingerprint in Table 1 to represent single fingerprint attributes, such as ecfp2 represents the single fingerprint attribute extracted from ECFP2 fingerprint, and multiple single fingerprint attributes connected by ’_’ represent dual fingerprint attributes and triplet fingerprint attributes, such as ecfp0${\_}$ecfp2, hashap${\_}$avalon${\_}$ecfp4. For the deep attributes, we extract seven types of deep fingerprints by using seven self-supervised learning methods mentioned above, $\mathcal{S}=\left \{ \bigcup _{l=1}^{7} \mathcal{S}^{l} \right \}$ for all $n$ molecules, and reduce the dimension to 100 dimensions through PCA to obtain deep attributes, denoted as $\mathcal{D}=\phi (\mathcal{S})$ and $d_{i}^{l}\in \mathbb{R}^{100}$. In the following, we use ’CGIP_G’, ’GraphMVP’, ’IEM_3d_10conf’, ’MoleBERT’, ’molformer’, ’unimol_10conf’, ’VideoMol_1conf’ to represent deep attributes, respectively. ’1conf’ and ’10conf’ denote models using 1 and 10 conformers, respectively. Finally, we select any one attribute $a$ from $\left \{\mathcal{A}^{1},\mathcal{A}^{2},\mathcal{A}^{3},\mathcal{D}\right \}$ to guide the training and inferring of the model.

### AGDA module

Here, we incorporate the molecular attributes and design an AGDA module to learn more informative and discriminative molecular representations. The detailed structure of AGDA is illustrated in Fig. 2(c). AGDA consists of an attribute-guided local attention module and an attribute-guided global attention module, which guide the model to focus on important local information and global details, respectively.

First, all nodes representations are obtained by GAT for molecule $x_{i}$, denoted by $G_{i} = \{g_{j}\}_{j=1}^{j=N}\in \mathbb{R}^{d^{g}}$, where $d^{g}$ represents the length of the node representation and $N$ represents the number of nodes. The input of attribute-guided local attention module is $F_{l{\_}inp} = {[g_{j};a]}_{1}^{N}\in \mathbb{R}^{(d^{g}+d^{a})}$, where $a$ is the attributes of molecule $x_{i}$, $d^{a}$ is the length of attributes and [;] denotes the concatenation. Then, we use a fully connected layer with sigmoid function to compute the local attention, 


(1) \begin{align*}& Attn_{local} = \sigma(f_{local}(F_{l{\_}inp}))\in \mathbb{R}^{N\times (d^{g})},\end{align*}

where $\sigma $ denotes the sigmoid activation function and $f_{local}$ denotes the fully connected layer. To obtain the node representations refined by local attention, we multiply $Attn_{local}$ with the node representations $G_{i}$, expressed as 


(2) \begin{align*}& F_{l{\_}out} = Attn_{local} \otimes G_{i} = \{g_{j}^{\prime}\}_{1}^{N}\in \mathbb{R}^{d^{g}},\end{align*}

where $F_{l{\_}out}\in \mathbb{R}^{N\times d^{g}}$ represents the output of the local attention module and $ \otimes $ denotes element-wise multiplication.

For the attributes-guided global attention module, we first get the representation of molecule $x_{i}$ by averaging all node representations, $g_{i} = \frac{1}{N} \sum _{j}^{N}g_{j}^{\prime}\in \mathbb{R}^{d^{g}}$. The input of the module is $F_{g{\_}inp} = [g_{i};a]\in \mathbb{R}^{d^{g}+d^{a}}$. We also use a fully connected layer and sigmoid function to obtain global attention, which can be formulated as follows: 


(3) \begin{align*}&Attn_{global} = \sigma(f_{global}(F_{g{\_}inp})\in \mathbb{R}^{d^{g}},\end{align*}

Finally, we multiply $Attn_{global}$ with $g_{i}$ to obtain the final refined molecular representation and formalize as follows: 


(4) \begin{align*}& F_{g{\_}out} = Attn_{global} \otimes g,\end{align*}

where $F_{g{\_}out}\in \mathbb{R}^{d^{g}}$ is the final molecular representation refined by molecular attributes.

### Training and evaluation

APN is based on prototype network, which means that a prototypes for every class in a few-shot classification task need to be calculated. The attribute-refined molecular representations in a task after going through the AGDA module is denoted as $Z_{t}^{\prime} = \{\mathbf{z}^{\prime}_{i}\}_{i=1}^{2K+q}\in \mathbb{R}^{100}$. The prototype representations of the positive (negative) examples, $\mathbf{p}_{positive}$ ($\mathbf{p}_{negative}$), is computed by the weighted sum of all the positive (negative) examples. Specifically, for each embedded support point within a class, we compute a distance, which represents the sum of Euclidean distances between it and the other points. The weight assigned is inversely proportional to the distance; larger distances result in smaller weights. Formulately, the positive prototype is computed as follows: 


(5) \begin{align*}& \begin{cases} \mathbf{p}_{positive} = \sum_{i}^{K}a_{i}\mathbf{z}^{\prime}_{i},\,\quad\qquad i \epsilon [1,K] \\[5pt] a_{i} = weight_{i} / \sum_{j}^{K}weight_{j},\, j \epsilon [1,K]\\[5pt] weight_{i} = 1 / distance:{i},\\[5pt] distance:{i} = \sum_{j}^{K}\mathbf{L}2(\mathbf{z}^{\prime}_{i},\mathbf{z}^{\prime}_{j}),\, j \epsilon [1,K]\\ \end{cases}\end{align*}

The label of the molecule in the query set is determined by calculating the dot product similarity between it and the two prototypes. During the meta-training process, the predicted labels are used to calculate the loss for updating the model parameters: 


(6) \begin{align*}& \begin{cases} L_{i} = - [y_{i}\cdot log(p_{i})+(1-y_{i})\cdot log(1-p_{i})],\\[5pt] Loss_{t} = \frac{1}{q} \sum_{i}^{q}L_{i},\, i \in [1,q]\\ \end{cases}\end{align*}

Here, $y_{i}$ represents the label of molecule $i$, with the positive class denoted as 1 and the negative class as 0. $p_{i}$ represents the probability that molecule $i$ is predicted to be a positive sample. During meta-testing process, the predicted label for the target task is used to determine the activity of a molecule. Algorithm 1 shows the specific algorithm details of APN. 


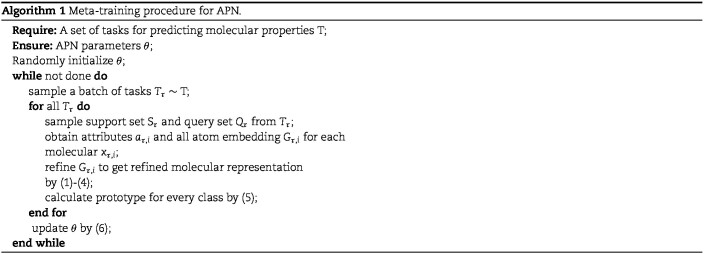


## Experiment

### Experimental settings

**Datasets.** We validate our method on three widely used few-shot MPP datasets from MoleculeNet [69] and follow the data splits in [25]. Details of the three datasets are shown in Table 2, which includes the number of tasks, the division of meta-training and meta-testing tasks, and number of molecules.

- Tox21 (https://tripod.nih.gov/tox21/challenge/) contains toxicity information of 7831 molecules in 12 assays (each assay corresponds to a specific target), among which 9 assays are split for training and 3 assays are split for testing.
- SIDER [70] records the side effects information of 1427 compounds, where 5868 side effects are grouped into 27 categories as in [1], among which 21 categories are split for training and 6 categories are split for testing.
- MUV [2] is a challenging virtual screening dataset, containing 93 127 compounds in 17 assays, among which 12 assays are split for training and 5 assays are split for testing.

**Table 2:** The detail information of datasets

Dataset	Tox21	SIDER	MUV
Compounds	7831	1427	93 127
Tasks	12	27	17
Meta-Training Tasks	9	21	12
Meta-Testing Tasks	3	6	5

**Details of molecular graphs.** To extract features from molecules, we uses RDKit [67] to construct molecular graphs from the raw SMILES sequences. In these graphs, we extract essential atom features, including atom number and chirality tags, as well as bond features such as bond type and bond direction. See Appendix Table 1 for more details about features of atoms and bounds. Finally, we employ a five-layer Graph Attention Network (GAT) to encode the information contained within the molecular graph and derive molecular and nodes embeddings.

**Implementation details.** We mainly use PyTorch to implement the APN framework and uses the Adam optimizer [71] with a learning rate ranging from 0.0005 to 0.05 for gradient descent optimization. GAT has five layers and the dimension of molecular is 100. The APN framework is trained on 4$\times $Tesla T4 GPUs with Intel(R) Xeon(R) Silver 4210R CPU @ 2.40 GHz on Ubuntu 18.04 platform. During training, 2000 episodes are generated in 2-way 10-shot during training. The cross entropy loss is used as the loss function of the classification task and we employ an early stop strategy with a patience level of 100 during model training. During the testing phase, following [16], a batch of support sets with size 10 or 20 and a batch of query sets with size 32 are randomly sampled from the test task. For each test task, 20 independent runs were performed based on different random seeds to mitigate randomness, and the average value of performance was calculated as the final performance.

**Evaluation protocol.** Following previous works [16, 23], we employed area under the receiver operating characteristic curve (ROC-AUC), F1 score, and precision-recall area under the curve (PR-AUC) calculated on the query set of meta-testing tasks to comprehensively evaluate the performance of our model and the comparison methods. We run experiments for three times and report the mean and standard deviations of ROC-AUC, F1 score, and PR-AUC across all meta-testing tasks for each compared method at the support set size 10 and 20 (i.e. 5- and 10-shot). For fair comparison, we used the same experimental settings and reproduced several common baselines (Siamese [72], AttnLSTM, IterRefLSTM [25], MetaGAT [16]) based on the source codes they provided. We did not perform 1-shot learning, as it is an unrealistic scenario in real-world drug discovery.

### Main results

**Performance comparison.** We compare APN with multiple baseline models under the same experimental setup, including Siamese, attention LSTM (attnLSTM), IterRefLSTM, and Meta-GAT. As shown in Tables 3, 4, and 5, APN consistently outperforms all other models in most cases with average improvements of 1.69% on ROC-AUC, 1.65% on F1 score, and 1.89% on PR-AUC, demonstrating its effectiveness.

**Table 3:** ROC-AUC scores with standard deviations of all compared methods on Tox21 dataset. The best results are highlighted in bold font.

Method	5-shot			10-shot		
	ROC-AUC	F1-Score	PR-AUC	ROC-AUC	F1-Score	PR-AUC
Siamese	63.34 (2.15)	55.34 (3.50)	64.28 (2.51)	70.71 (1.40)	57.98 (8.89)	71.35 (1.44)
AttnLSTM	58.69 (1.69)	49.62 (4.16)	58.58 (2.31)	65.97 (3.80)	56.71 (7.25)	65.87 (3.31)
IterRefLSTM	75.09 (2.25)	66.54 (2.59)	74.02 (2.21)	74.46 (0.21)	61.28 (4.94)	73.41 (0.90)
MetaGAT	79.98 (0.11)	74.03 (0.51)	78.73 (0.46)	82.40 (1.00)	74.70 (1.81)	82.36 (0.94)
APN	**80.40 (0.23)**	**74.04 (0.55)**	**79.84 (0.41)**	**84.54 (0.36)**	**76.16 (0.79)**	**84.86 (0.59)**

**Table 4:** ROC-AUC scores with standard deviations of all compared methods on SIDER dataset. The best results are highlighted in bold font.

Method	5-shot			10-shot		
	ROC-AUC	F1-Score	PR-AUC	ROC-AUC	F1-Score	PR-AUC
Siamese	52.69 (0.29)	32.56 (9.62)	52.45 (0.86)	55.86 (0.93)	29.97 (5.55)	56.07 (1.38)
AttnLSTM	49.51 (0.84)	41.73 (5.27)	54.54 (2.14)	49.18 (2.52)	35.41 (6.85)	53.50 (3.11)
IterRefLSTM	66.52 (2.40)	65.11 (2.55)	65.57 (2.22)	63.19 (2.23)	55.21 (10.03)	62.44 (1.72)
MetaGAT	**77.31 (0.20)**	**71.97 (0.68)**	**76.45 (0.45)**	77.73 (0.72)	71.05 (1.63)	77.22 (1.05)
APN	75.07 (0.38)	69.16 (1.35)	74.36 (0.54)	**79.02 (0.72)**	**71.68 (1.79)**	**78.66 (0.6)**

**Table 5:** ROC-AUC scores with standard deviations of all compared methods on MUV dataset. The best results are highlighted in bold font.

Method	5-shot			10-shot		
	ROC-AUC	F1-Score	PR-AUC	ROC-AUC	F1-Score	PR-AUC
Siamese	49.94 (0.73)	33.32 (6.73)	54.97 (2.84)	49.59 (0.86)	33.56 (1.64)	57.90 (2.88)
AttnLSTM	50.74 (0.49)	32.84 (3.43)	53.31 (2.65)	50.99 (0.21)	29.65 (3.18)	54.80 (4.93)
IterRefLSTM	50.95 (11.85)	53.30 (10.50)	50.44 (2.91)	54.11 (13.82)	56.51 (7.21)	51.74 (4.32)
MetaGAT	65.21 (1.32)	59.91 (1.49)	63.10 (1.52)	65.22 (0.84)	57.02 (4.09)	63.97 (0.59)
APN	**68.35 (0.25)**	**60.82 (0.53)**	**67.31 (1.03)**	**70.63 (0.80)**	**66.72 (1.34)**	**68.11 (1.14)**

**Figure 3:**
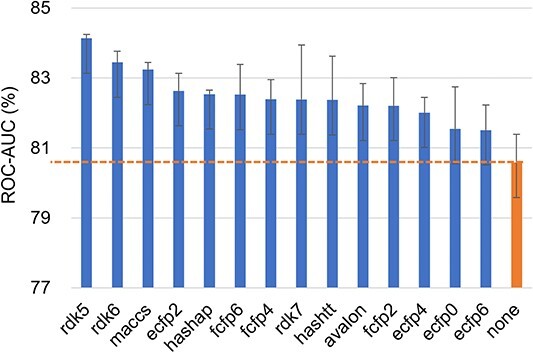
The ROC-AUC score of APN with 14 single fingerprint attributes on 10-shot tasks from Tox21 dataset. ’none’ is the result of APN when no attribute is used.

**Figure 4:**
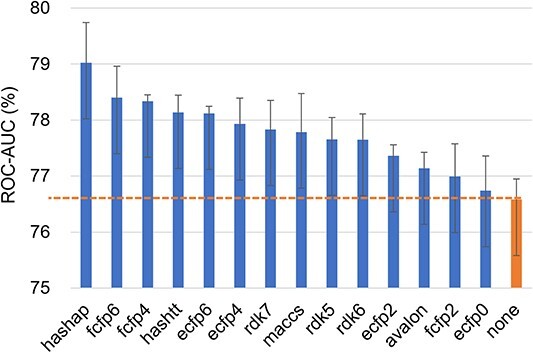
The ROC-AUC score of APN with 14 single fingerprint attributes on 10-shot tasks from SIDER. ’none’ is the result of APN when no attribute is used.

**Figure 5:**
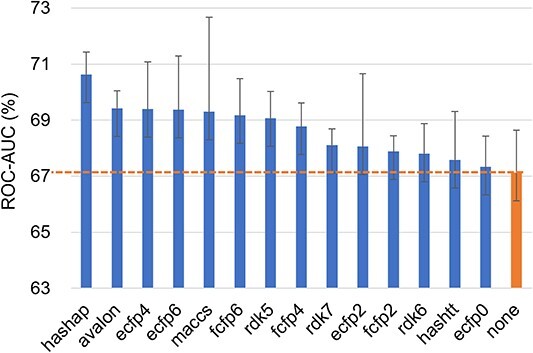
The ROC-AUC score of APN with 14 single fingerprint attributes on 10-shot tasks from MUV. ’none’ is the result of APN when no attribute is used.

**Single fingerprint attributes.** We conduct a comprehensive study to investigate the impact of single fingerprint attributes on FS-MPP across the three datasets mentioned above. The experimental results of APN with different single fingerprint attributes on the Tox21, SIDER, and MUV datasets are shown in the Figs 3, 4, and 5, which illustrates that the use of single fingerprint attributes leads to significant performance improvements compared to the results without attributes (denoted as ’none’), with a maximum improvement of 3.55, 2.32, and 2.77%, respectively. Notably, we observe that path-based fingerprint attributes, such as rdk5, rdk6, and hashap, significantly contribute to performance improvement. For a more comprehensive presentation of the experimental results, please refer to the Appendix Table S3. Additionally, we explored the effects of dimensionality reduction through K-Means clustering [73], the details can be found in the Appendix Table S2.

**Dual fingerprint attributes.** To investigate whether combining more fingerprint information into molecular attributes would further improve prediction performance and the relationships between single fingerprint attributes, we combine 14 single fingerprint attributes in pairs to get dual fingerprint attributes and define three types of relationships: mutual promotion (R1): $AUC_{fp1+fp2}>AUC_{fp1} \ and \ AUC_{fp1+fp2}>AUC_{fp2}$, one-sided promotion (R2): $AUC_{fp1+fp2}> AUC_{fp1} \ or \ AUC_{fp1+fp2} > AUC_{fp2}$, and mutual inhibition (R3): $AUC_{fp1+fp2} < AUC_{fp1} \ and \ AUC_{fp1+fp2} < AUC_{fp2}$, where $AUC_{fp1+fp2}$, $AUC_{fp1}$ and $AUC_{fp2}$ represent the ROC-AUC score of APN with dual fingerprint attribute obtained by combining fp1 attribute and fp2 attribute, APN with fp1 attribute, and APN with fp2 attribute, respectively.

We combine 14 single fingerprint attributes in pairs through addition or concatenation, and conduct experiments on the 10-shot tasks from the Tox21 dataset. The top 10 ROC-AUC scores for the two combinations are illustrated in Table 7. It can be observed that dual fingerprint attributes do not further enhance the peak ROC-AUC score. However, in comparison to single fingerprint attributes, dual fingerprint attributes are more stable, with all top 10 ROC-AUC score surpassing 0.835. For all experimental results, please refer to the Appendix Tables S4 and S5.

**Table 6:** The ROC-AUC score of APN that uses deep attributes on 10-shot tasks from Tox21, SIDER, and MUV datasets.

Attributes	Tox21		SIDER		MUV		Average
	5-shot	10-shot	5-shot	10-shot	5-shot	10-shot	
CGIP_G	78.81 (0.46)	81.77 (0.07)	72.77 (0.53)	78.69 (0.30)	67.18 (1.17)	67.33 (0.22)	74.43
GraphMVP	78.19 (0.37)	81.52 (0.17)	73.00 (0.83)	76.86 (0.35)	66.26 (0.43)	66.66 (0.23)	73.75
IEM_3d_10conf	79.79 (0.30)	82.70 (0.55)	71.50 (0.80)	77.53 (0.41)	66.68 (1.61)	**69.23 (1.32)**	74.57
MoleBERT	80.40 (0.23)	**84.54 (0.36)**	73.20 (0.28)	78.58 (0.23)	66.48 (0.24)	67.35 (0.16)	75.09
molformer	79.60 (0.36)	82.26 (0.13)	73.19 (1.04)	**79.05 (0.23)**	66.35 (0.40)	67.30 (0.42)	74.63
unimol_10conf	**80.66 (0.91)**	84.21 (0.62)	**73.57 (0.72)**	77.08 (1.45)	66.57 (0.22)	67.13 (0.46)	74.87
VideoMol_1conf	79.52 (0.34)	83.66 (0.65)	73.34 (0.78)	78.81 (0.29)	**67.39 (0.58)**	68.17 (0.18)	**75.15**

**Table 7:** The top 10 ROC-AUC results of APN with dual fingerprint attributes on 10-shot tasks from Tox21.

addition		concatenation	
Attribute	ROC-AUC	Attribute	ROC-AUC
ecfp4_rdk5	84.19	ecfp2_rdk5	84.19
rdk5_hashtt	84.12	ecfp2_maccs	83.95
fcfp2_rdk5	84.02	fcfp4_rdk5	83.89
ecfp0_rdk5	83.95	fcfp2_rdk5	83.85
fcfp6_rdk5	83.95	fcfp2_hashap	83.85
ecfp0_maccs	83.82	ecfp0_rdk6	83.81
fcfp6_hashtt	83.74	ecfp4_avalon	83.74
ecfp6_rdk5	83.62	fcfp2_rdk7	83.72
fcfp2_rdkDes	83.57	ecfp4_rdk5	83.72
ecfp4_rdk6	83.56	fcfp4_hashap	83.70

**Figure 6:**
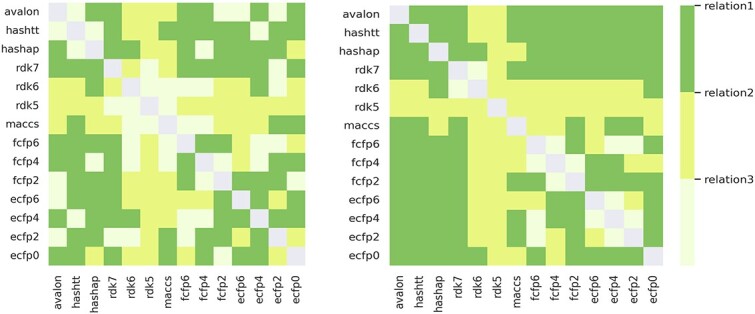
Heatmap of relationships between two single fingerprint attributes. The left shows the result of the APN with dual fingerprint attributes obtained by summing two single fingerprint attributes, while the right shows the result of the APN with dual fingerprint attributes obtained by concatenating two single fingerprint attributes.

We generate a tricolored heatmap to gain a more intuitive understanding of the relationship between single fingerprint attributes. Specifically, if the relationship between two attributes is R1, the corresponding value in the heatmap is 1; R2 and R3 are 0.5 and 0, respectively. The heatmaps are presented in Fig. 6. It is noticeable that circular-based fingerprints and path-based fingerprint often exhibit a mutual reinforcing relationship; more than half of the relations belong to R1. In view of the above results, we further study whether triplet fingerprint attributes can further improve the prediction performance.

**Triplet fingerprint attributes.** Considering that the combination space of three different single attributes is too large, we only consider dual fingerprint attributes that the two attributes in it are mutually prompting (R1). Then, we use these selected dual fingerprint attributes to further combine with a single fingerprint attributes through addition to obtain triplet fingerprint attributes. We conducted experiments on the 10-shot tasks from the Tox21 dataset and show the top 20 triplet fingerprint attributes with the highest performance in Table 8. Compared with all the single fingerprint and dual fingerprint attributes, the ’hashap_avalon_ecfp4’ attributes exhibite a slight improvement. Furthermore, we observe that the more fingerprint information is used, the better the performance (’hashap_avalon_ecfp4’: 0.8446, ’hashap_avalon’: 0.8352, ’ecfp4_hashap’: 0.8291, ’ecfp4_avalon’: 0.8258, ’hashap’: 0.8254, ’avalon’: 0.8221, ’ecfp4’: 0.8201). This indicates a synergistic relationship among these three fingerprints, highlighting that combining multiple fingerprint attributes can indeed enhance performance. However, since the performance improvement is relatively slight, we recommend using single fingerprint attributes to enhance efficiency and conserve resources.

**Table 8:** The top 20 ROC-AUC result of APN with triplet fingerprint attributes on 10-shot tasks from Tox21.

Triplet fingerprint attributes	ROC-AUC
hashap_avalon_ecfp4	84.46
hashap_avalon_fcfp2	84.33
fcfp2_hashap_rdk6	84.32
rdk7_hashap_avalon	84.23
ecfp6_hashap_ecfp2	84.22
hashap_avalon_rdk7	84.19
rdk6_hashap_avalon	84.12
ecfp2_ecfp4_rdk5	84.04
fcfp2_fcfp6_rdk5	84.03
ecfp6_fcfp2_rdk5	84.02
ecfp4_hashap_ecfp6	84.00
rdk6_hashap_fcfp4	83.99
ecfp2_hashap_avalon	83.98
fcfp2_hashap_avalon	83.98
rdk7_avalon_ecfp6	83.97
rdk6_hashap_rdk7	83.96
ecfp0_fcfp4_hashtt	83.93
hashap_avalon_fcfp6	83.93
ecfp4_rdk7_avalon	83.90
ecfp0_hashtt_fcfp4	83.90

**Deep attributes.** We study the performance of APN with seven deep attributes, i.e. molecular attributes directly obtained from the sequence, graphs, and images through self-supervised learning methods. The experimental results on Tox21, SIDER, and MUV datasets is shown in Table 6, which shows that the performance of deep attributes is comparable to that of fingerprint attributes, and even performs better than fingerprint attributes on the Tox21 dataset.

### Ablation Study

**Figure 7:**
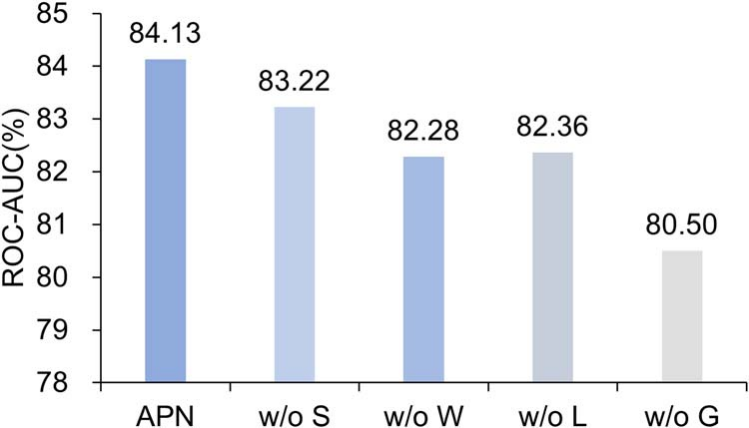
Ablation study on 10-shot tasks from Tox21.

**The effectiveness of APN modules.** We implement four variants of APN to show the effectiveness of modules in APN, including: (i) w/o L: w/o applying the attributes-guided local-attention module; (ii) w/o G: w/o applying the attributes-guided global-attention module; (iii) w/o S: w/o applying the dot product similarity, i.e. using L2 distance ; (v) w/o W: w/o applying weighted sum when calculating prototypes. The results on 10-shot tasks from Tox21 are depicted in Fig. 7. APN obtains better performance than its variants, demonstrating that the components in the APN can effectively collaborate to improve performance. There are several findings from these experimental results. First, w/o G has the worst performance in all cases, illustrating the crucial ability of attributes-guided global-attention module to capture information related to specific few-shot MPP tasks. Second, attributes-guided local-attention module in APN can significantly improve performance compared to without it (w/o L), demonstrating its effectiveness. However, the performance gain of attributes-guided local-attention module is slightly worse than that of attributes-guided global-attention module, indicating that molecular attribute information is more suitable for guiding global information. Third, APN outperforms w/o S and w/o W, demonstrating the benefit of incorporating dot product similarity and weighted prototypes into APN.

**Figure 8:**
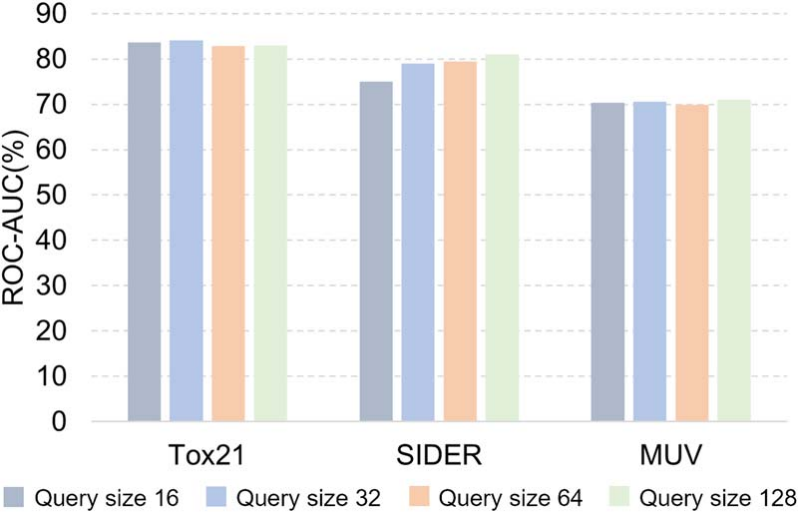
Ablation study on query size.

**Different query set sizes.** In order to verify whether the query set size has an impact on the performance of APN, we conduct an ablation study using different query set sizes (16, 32, 64, 128) for comparison. The experimental results on Tox21, SIDER and MUV datasets are shown in Fig. 8. It can be found that under different query sizes, the performance of APN is robust in most cases.

### Using Other Graph-based Molecular Encoders

**Figure 9:**
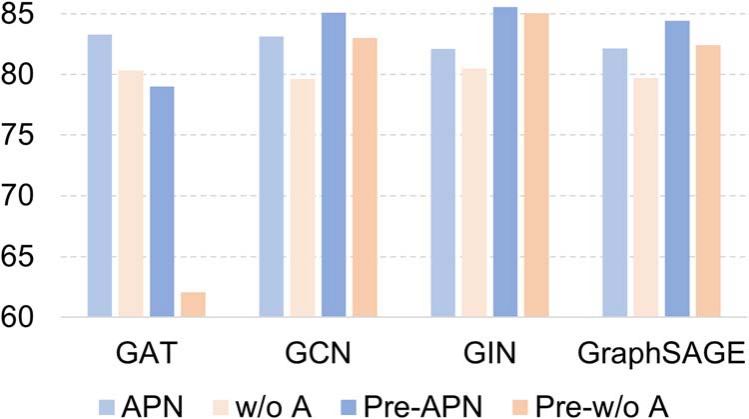
ROC-AUC scores (%) of APN on 10-shot tasks from Tox21 using different graph-based molecular encoders.

As introduced in Section 5, the molecular encoder GAT we used in the above experiment can be replaced by other graph-based molecular encoders. Here we consider three other graph-based molecular encoders: GCN, GIN and GraphSAGE, which are either learned from scratch or pretrained. Figure 9 shows the ROC-AUC scores on Tox21. It can be seen that GAT performs best among those learned from scratch. APN outperforms the w/o A (APN without attributes and AGDA) consistently, indicating the effectiveness of the molecular attributes and AGDA module of APN. We further notice that using pretrained encoders can improve the performance except for GAT, which is also observed in [74].

### Generalization ability verification

In order to verify the generalization ability of APN, we selected all the classification tasks in the TDC platform to construct the TDC dataset. Three absorption datasets, one distribution dataset, and three metabolism datasets in the TDC platform are used for meta-training, and three toxicity datasets are used for meta-testing. The detailed of the TDC dataset is shown in the Table 9. The training and test data in the TDC dataset belong to different domains, which can test the generalization ability of APN across these domains. The performance of Meta-GAT (best comparison method) and APN on 5- and 10-shot tasks is shown in the Table 10. Experimental results demonstrate that APN remains robust on unseen domains and outperforms Meta-GAT in both 5- and 10-shot tasks with an average improvement of 6.98% on AUC, 2.97% on F1-Score, and 5.34% on PR-AUC.

**Table 9:** The detail information of TDC datasets

No.	Dataset	Sample	Type
1	hia_hou	578	Absorption
2	pgp_broccatelli	1218	
3	bioavailability_ma	640	
4	bbb_martins	2030	Distribution
5	cyp2c9_substrate_carbonmangels	669	Metabolism
6	cyp2d6_substrate carbonmangels	667	
7	cyp3a4_substrate carbonmangels	670	
8	herg	655	Toxicity
9	ames	7278	
10	dili	475	

**Table 10:** ROC-AUC scores with standard deviations of all compared methods on TDC dataset. The best results are highlighted in bold font.

Method	5-shot		10-shot			
	AUC	F1-Score	PR-AUC	AUC	F1-Score	PR-AUC
Meta-GAT	62.78 (1.57)	63.40 (3.89)	62.61 (0.22)	64.26 (2.57)	60.26 (2.40)	64.66 (2.62)
APN	**68.35 (1.10)**	**63.67 (1.65)**	**67.21 (1.10)**	**72.65 (0.28)**	**65.92 (1.55)**	**70.74 (0.78)**

## Conclusion

In this work, we propose a novel attributes-guided framework called APN to address the challenge of FS-MPP. APN extracts molecular attributes and designs an AGDA module to learn the relationship between the graphs and attributes. Unlike common FS-MPP methods that solely rely on the structural information of molecules, We utilize 14 types of molecular fingerprints and 7 types of deep fingerprints to obtain molecular attributes, which encapsulate high-level molecular knowledge defined by experts and self-supervised learning methods to guide deep neural networks in learning molecules. The experiments on benchmark datasets validate the effectiveness and generalization ability of APN. Furthermore, we discover that path-based fingerprints perform the best, such as rdk5, rdk6, hashap, and hashtt; among circular-based fingerprints, ecfp4, ecfp6, fcfp4, and fcfp6 perform relatively well; in the category of substructure-based fingerprints, maccs often outperforms avalon, but it may have greater variance. In the future, we plan to explore more molecular attributes, such as textual descriptions, knowledge graph and knowledge predicted by models, for learning molecular representations in data-scarce scenarios.

**Key Points**

- We propose an attribute-guided prototype network (APN) for few-shot molecular property prediction (FS-MPP), which leverages human-defined high-level attributes to guide the model to specifically learn key features related to molecular properties.
- Considering that high-level attribute knowledge can guide the few-shot model to learn molecules more effectively and accurately, and the molecular fingerprint and self-supervised learning methods can provide a large number of information related to molecular properties such as chemical structure, physicochemical properties and characteristics, so we design an attribute extractor to extract the fingerprint attributes from 14 kinds of molecular fingerprints and deep attributes from self-supervised learning methods to guide the learning of APN.
- We alleviate an attribute-guided dual-channel attention module (AGDA), which refines atomic-level and molecular-level representations through local attention and global attention, allowing the model to learn the mapping between representations and high-level attribute concepts.
- The experimental result on multiple benchmark datasets and ablation experimental results show the effectiveness of AGDA module of APN and that the guidance of molecular attribute is greatly beneficial to FS-MPP tasks. In addition, the strong generalization ability of APN is verified by conducting experiments on data from different domains.
- We study different molecular attributes extracted from fingerprints or self-supervised learning methods, compared and analyzed the performance of these attributes on FS-MPP tasks, and visualized the relationships between different attributes according to the performance, providing insights for future studies of molecular attributes.

## Supplementary Material

Appendix_bbae394

## Data Availability

The authors declare no competing financial interest. All of the data used for the validation of this study (Tox21, SIDER, and MUV) are publicly available. The data and code are freely available at https://github.com/hou29/few-shot-MPP.
