# Supplementary material for: Attribute-guided prototype network for few-shot molecular property prediction
Source: Brief Bioinform. 2024 Aug 12;25(5):bbae394. doi: 10.1093/bib/bbae394 (PMC11318080; doi:10.1093/bib/bbae394)
Supplement: Appendix_bbae394 [file appendix_bbae394.pdf]

Appendix A. The Features of Molecular Grpah

We extract essential atom features, including atom number and chirality tags, and bond features such as bond type and bond direction. Table S1 describe the details of the features of atoms and bonds.

Table S1: The features of atoms and bonds.

Feature	Size	Description
atom type	119	atomic type (i.e., C, N, O)
chirality tag	4	chirality type (i.e., clockwise R type, counter Clockwise S type)
bond type	4	bound type (i.e., single, double, triple, aromatic)
bond direction	3	bond direction(none, endupright, enddownright)

Appendix B. Molecular Fingerprint Details

We selected 14 fingerprints of the following 3 different types. Ring-based fingerprints: Ring-based fingerprints include extended connection fingerprints (ECFPx) and functional class fingerprints (FCFPx), where x represents the bond length or diameter centered on the atom. The x values of ECFP and FCFP are 0, 2, 4, 6 and 2, 4, 6 respectively. ECFP fingerprints include information such as the number of atomic connections, the number of non-hydrogen chemical bonds, the atomic number, the positive and negative atomic charge, the absolute value of the atomic charge, and the number of connected hydrogen atoms. FCFP mainly contains information on pharmacophores, such as hydrogen bond acceptors, hydrogen bond donors, negatively ionizable, positively ionizable, aromatic atoms, and halogens. Path-based fingerprints: Path-based fingerprints include RDKx (x is 5, 6, or 7), HashTT (topological torsion), and HashAP (atom pairs). HashTT is similar to HashAP and contains information in three dimensions: atomic number, number of φ electron, and number of adjacent atom. Substructure-based fingerprints: Substructure-based fingerprints include MACCS (Molecular ACCess System) and Avalon. MACCS is a molecular fingerprint with a length of 167, and each bit represents a SMARTS-encoded substructure. Avalon fingerprints include features such as atomic symbol path, atom count, augmented symbol path, and augmented atom.

Appendix C. Experimental Details

Specific definitions and sizes of attributes. For the fingerprint attributes, we first extract 14 single fingerprint attributes from 14 fingerprints for all molecules in datasets. Here, we use PCA technology to reduce the dimensions of 14 fingerprints to 100 dimensions to obtain the corresponding 14 single fingerprint attributes with 100 dimensions. Then, we get dual fingerprint attributes and triplet fingerprint attributes by concatenating or summing two or three single fingerprint attributes. So the dimension of dual fingerprint attributes can be 100 or 200; the dimension of triplet fingerprint attributes can be 100 or 300. We use the lowercase form of the fingerprint in Table 1 to represent single fingerprint attributes, such as ecfp2 represents the single fingerprint attribute extracted from ECFP2 fingerprint, and multiple single fingerprint attributes connected by '_' represent dual fingerprint attributes and triplet fingerprint attributes, such as ecfp0_ecfp2, hashap_avalon_ecfp4.

For the deep attributes, we automatically extract 7 types of deep fingerprints from 7 self-supervised learning methods mentioned above for all molecules in datasets, and reduce the dimension to 100 dimensions through PCA to obtain deep attributes with 100 dimensions. We use 'CGIP_G', 'GraphMVP', 'IEM_3d_10conf', 'MoleBERT', 'molformer', 'unimol_10conf', 'VideoMol_1conf' to represent deep attributes respectively. Finally, we select any one attribute from single fingerprint attributes, dual fingerprint attributes, triplet fingerprint attributes and deep attributes to guide the training and inferring of the model.

Hyperparameter settings. In all experiments, we use a 5-layer GAT to encode molecules, with a dropout ratio of 0.2. The dimensionality of the molecular representation output by GAT is 100, and the dimensionality of the molecular attributes is 100. The learning rate is selected in [0.0005, 0.001, 0.005, 0.01, 0.05].

Training and inference details. In the training process, GAT is firstly used to extract nodes representation from molecules in support set and query set. Then, the selected molecular attribute (generated directly by the attribute extractor or loaded from a pre-saved attribute file) and the nodes representation of the molecules are fed into the AGDA module. In AGDA, molecular attributes are used to refine atomic-level and molecular-level representation through local and global attention to make the representation of molecules more informative and discriminative. Finally, the prototypes of positive and negative samples are separately calculated in a weighted manner. The label of the molecule in the query set is determined by calculating the dot product similarity between it and the two prototypes. The cross entropy loss function is used to calculate the loss. The loss of a training task is the average of the sum of the losses of all samples in the query set. The loss of all training tasks are added together and back-propagated to update the model parameters.

During inference, as in the training process, two prototypes are first calculated. To predict the properties of a new molecule, we only need to calculate the dot product similarity between it and the two prototypes. The one with the higher similarity is the label of the molecule.

Appendix D. More Experimental Results

To investigate the impact of different dimensionality reduction methods on performance, we also employ clustering to reduce the dimensionality of molecular fingerprints on the tox21 dataset and adjusted the learning rate to obtain the experimental results shown in Table S2 below. It can be observed that the performance is not as good as that achieved with PCA. We believe this is because the information obtained after dimensionality reduction through PCA is richer.

We present the experimental results of APN with single fingerprint attributes on the Tox21, SIDER, and MUV datasets in Table S3. The results without any guidance of fingerprint attributes are marked as none.

To investigate whether the combination of multiple fingerprint information can improve the performance of few-shot molecular property prediction, we combine two different molecular fingerprint information as the attributes of the molecules. We consider two ways of combining fingerprint information: summation or concatenation, and the experimental results of these two combination methods on the Tox21 dataset are shown in Table S4 and Table S5.

Table S2: The AUC result on 10-shot tasks from Tox21 of dimensionality reduction through clustering.

Fingerprint	lr	AUC(%)	Fingerprint	lr	AUC(%)	Fingerprint	lr	AUC(%)
hashtt	0.005	82.52	hashtt	0.001	81.73	rdk5	0.0005	81.22
hashap	0.005	82.18	ecfp2	0.001	81.73	fcfp2	0.0005	81.20
ecfp0	0.005	81.96	fcfp6	0.001	81.69	hashap	0.0005	81.05
fcfp4	0.005	81.90	ecfp6	0.001	81.65	fcfp4	0.0005	80.99
fcfp6	0.005	81.79	rdk5	0.001	81.58	rdk7	0.0005	80.95
rdk5	0.005	81.62	hashap	0.001	81.51	avalon	0.0005	80.94
ecfp2	0.005	81.59	ecfp0	0.001	81.38	ecfp6	0.0005	80.89
ecfp6	0.005	81.43	maccs	0.001	81.29	maccs	0.0005	80.87
maccs	0.005	81.35	rdk7	0.001	81.25	rdk6	0.0005	80.86
rdk7	0.005	81.32	fcfp2	0.001	81.21	fcfp6	0.0005	80.79
rdk6	0.005	81.20	rdk6	0.001	81.16	ecfp0	0.0005	80.76
avalon	0.005	81.02	fcfp4	0.001	81.15	ecfp2	0.0005	80.62
ecfp4	0.005	80.86	ecfp4	0.001	80.97	hashtt	0.0005	80.39
fcfp2	0.005	80.41	avalon	0.001	80.67	ecfp4	0.0005	80.25
none	0.005	79.73	none	0.001	80.56	none	0.0005	79.42

Table S3: The ROC-AUC score of APN with single fingerprint attributes on 2-way 10-shot tasks.

Tox21		SIDER		MUV	
Fingerprint	AUC(%)	Fingerprint	AUC(%)	Fingerprint	AUC(%)
rdk5	84.13	hashap	79.02	hashap	70.63
rdk6	83.45	fcfp6	78.40	avalon	69.42
maccs	83.24	fcfp4	78.34	ecfp4	69.39
ecfp2	82.63	hashtt	78.14	ecfp6	69.37
hashap	82.54	ecfp6	78.12	maccs	69.30
fcfp6	82.52	ecfp4	77.93	fcfp6	69.17
fcfp4	82.39	rdk7	77.83	rdk5	69.07
rdk7	82.38	maccs	77.79	fcfp4	68.77
hashtt	82.37	rdk5	77.66	rdk7	68.11
avalon	82.21	rdk6	77.65	ecfp2	68.05
fcfp2	82.21	ecfp2	77.36	fcfp2	67.88
ecfp4	82.01	avalon	77.14	rdk6	67.80
ecfp0	81.55	fcfp2	76.99	hashtt	67.58
ecfp6	81.51	ecfp0	76.74	ecfp0	67.33
none	80.58	none	76.58	none	67.13

Table S4: The ROC-AUC score (%) of APN when concatenating two single fingerprint attributes on 2-way 10-shot tasks from Tox21 dataset.

fingerprint	avalon	hashtt	hashap	rdk7	rdk6	rdk5	maccs	fcfp6	fcfp4	fcfp2	ecfp6	ecfp4	ecfp2	ecfp0
avalon	-	82.42	83.07	83.51	83.04	83.36	83.65	83.69	83.25	82.48	82.91	83.74	82.77	82.83
hashtt	82.42	-	83.40	83.04	83.40	83.65	83.29	83.37	83.33	82.80	82.68	83.09	83.16	83.10
hashap	83.07	83.40	-	83.15	83.65	83.37	83.21	82.91	83.70	83.85	82.74	83.33	83.51	83.14
rdk7	83.51	83.04	83.15	-	82.38	83.08	83.60	83.23	83.15	83.72	82.94	83.32	83.02	83.25
rdk6	83.04	83.40	83.65	82.38	-	83.56	83.38	83.18	83.07	83.40	83.21	83.03	82.93	83.81
rdk5	83.36	83.65	83.37	83.08	83.56	-	83.54	83.63	83.89	83.85	83.33	83.72	84.19	82.63
maccs	83.65	83.29	83.21	83.60	83.38	83.54	-	82.93	83.01	83.37	83.17	83.51	83.95	83.19
fcfp6	83.69	83.37	82.91	83.23	83.18	83.63	82.93	-	81.48	83.20	81.58	81.71	82.26	83.34
fcfp4	83.25	83.33	83.70	83.15	83.07	83.89	83.01	81.48	-	81.70	82.73	83.08	82.58	81.83
fcfp2	82.48	82.80	83.85	83.72	83.40	83.85	83.37	83.20	81.70	-	82.39	82.56	83.05	82.24
ecfp6	82.91	82.68	82.74	82.94	83.21	83.33	83.17	81.58	82.73	82.39	-	81.37	82.35	81.83
ecfp4	83.74	83.09	83.33	83.32	83.03	83.72	83.51	81.71	83.08	82.56	81.37	-	81.79	82.23
ecfp2	82.77	83.16	83.51	83.02	82.93	84.19	83.95	82.26	82.58	83.05	82.35	81.79	-	82.75
ecfp0	82.83	83.10	83.14	83.25	83.81	82.63	83.19	83.34	81.83	82.24	81.83	82.23	82.75	-

Table S5: The ROC-AUC score (%) of APN when summing two single fingerprint attributes on 2-way 10-shot tasks from Tox21 dataset.

fingerprint	avalon	hashtt	hashap	rdk7	rdk6	rdk5	maccs	fcfp6	fcfp4	fcfp2	ecfp6	ecfp4	ecfp2	ecfp0
avalon	-	82.15	83.52	83.20	83.37	83.04	82.54	82.81	82.57	81.57	81.40	82.58	81.90	82.45
hashtt	82.15	-	82.23	82.61	83.43	84.12	83.27	83.74	82.72	82.42	82.78	81.86	82.85	82.80
hashap	83.52	82.23	-	83.23	83.55	83.41	83.19	82.71	81.26	82.89	83.10	82.91	82.88	82.25
rdk7	83.20	82.61	83.23	-	82.53	82.29	82.58	83.14	83.11	82.96	83.51	82.88	82.01	83.07
rdk6	83.37	83.43	83.55	82.53	-	83.31	83.16	82.40	82.16	82.62	83.17	83.56	82.45	83.38
rdk5	83.04	84.12	83.41	82.29	83.31	-	82.76	83.95	83.02	84.02	83.62	84.19	83.14	83.95
maccs	82.54	83.27	83.19	82.58	83.16	82.76	-	82.11	81.29	83.08	81.75	82.63	83.30	83.82
fcfp6	82.81	83.74	82.71	83.14	82.40	83.95	82.11	-	83.32	82.53	82.14	81.18	81.60	82.36
fcfp4	82.57	82.72	81.26	83.11	82.16	83.02	81.29	83.32	-	81.57	82.21	81.61	83.21	82.83
fcfp2	81.57	82.42	82.89	82.96	82.62	84.02	83.08	82.53	81.57	-	83.07	82.78	83.29	80.26
ecfp6	81.40	82.78	83.10	83.51	83.17	83.62	81.75	82.14	82.21	83.07	-	82.66	82.09	82.36
ecfp4	82.58	81.86	82.91	82.88	83.56	84.19	82.63	81.18	81.61	82.78	82.66	-	82.82	82.43
ecfp2	81.90	82.85	82.88	82.01	82.45	83.14	83.30	81.60	83.21	83.29	82.09	82.82	-	82.42
ecfp0	82.45	82.80	82.25	83.07	83.38	83.95	83.82	82.36	82.83	80.26	82.36	82.43	82.42	-

Appendix E. Hyperparameter Sensitivity Analysis

To understand the robustness of APN to hyperparameters, we evaluated the impact of different learning rates and attribute dimensions on the performance of APN on Tox21, SIDER, and MUV datasets. We adjust the learning rate in the range of [0.0005, 0.001, 0.005, 0.01, 0.05, 0.1] and set the dimension of the attributes in the range of [100, 200, 300]. The experimental results are shown in the Fig S1. In the 10-shot results on the three datasets (the three lower figures in Fig. S1), we found that APN performs best with a learning rate of 0.005 and an attribute dimension of 300 on the Tox21 and SIDER datasets, while it achieves optimal performance on the MUV dataset with a learning rate of 0.001 and an attribute dimension of 100. Therefore, a moderate learning rate (0.001-0.005) and a larger attribute dimension(250-400) can further improve the results of APN. In the 5-shot results on the three datasets (the upper three figures in Figure S1), we found that the 5-shot results of the Tox21 dataset follow the same rule as the 10-shot results. On the SIDER dataset, the larger the attribute dimension, the better the performance, and the effect of the learning rate on APN is in the form of a bimodal curve (0.001 and 0.05). On the MUV dataset, the best performance is achieved when the attribute dimension is 300, and as the learning rate increases, the performance of APN gradually increases and reaches the maximum value at 0.1. Therefore, we suggest that the learning rate can be further increased to further improve the performance of APN on MUV dataset.

Figure S1: The ROC-AUC results with different learning rates and attributes dimensions on Tox21, SIDER and MUV datasets.

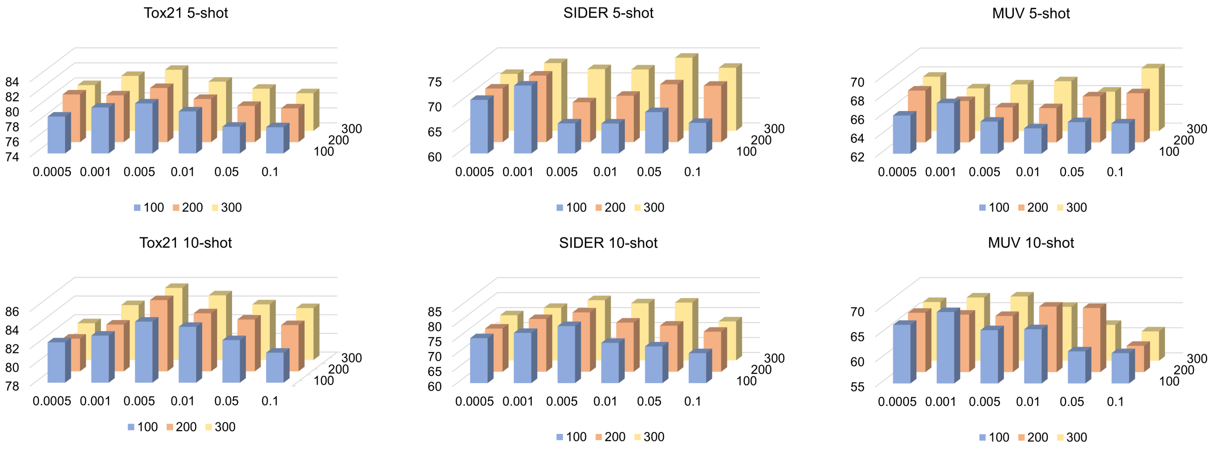

Appendix F. Time and Space Complexity

According to Fig.2a, APN mainly includes attribute extractor, molecular encoder (GNN), and attribute-guided dual-channel attention (AGDA) module. To analyze the time and space complexity of APN, we first analyze the time and space complexity of these modules one by one.

The AGDA module mainly includes the following operations: node attribute indexing, with a time complexity of O(N) and a space complexity of $O(N * attr\_dim)$, where N is the number of nodes and $attr\_dim$ is the dimension of attributes; concatenation of node features and attributes, with a time and space complexity of $O(N * (attr\_dim + emb\_dim))$, where emb_dim is the dimension of node embeddings; node weight calculation, with a time complexity of $O(N * emb\_dim * (emb\_dim + attr\_dim))$ and a space complexity of $O(N * (emb\_dim + attr\_dim))$; updating node representation with weights, with a time and space complexity of $O(N * emb\_dim)$; node embeddings aggregation, with a time complexity of $O(N * emb\_dim)$ and a space complexity of $O(M * emb\_dim)$, where M is the number of molecules; concatenating grpah features and attributes, with a time and space complexity of $O(M*(emb\_dim+attr\_dim))$; graph weight calculation, with a time complexity of $O(M*emb\_dim*(emb\_dim+attr\_dim))$ and a space complexity of $O(emb\_dim * (emb\_dim + attr\_dim))$; updating graph representation with weights, with a time and space complexity of $O(M * emb\_dim)$. Therefore, the total time and and space complexity of AGDA are: $O(N * emb\_dim * (emb\_dim + attr\_dim))$, $O(N * (emb\_dim + attr\_dim))$ respectively.

GNN mainly includes edge feature updates and message passing operations, with a time and space complexity of $O((E + N))$, where E and N represent the number of edges and nodes respectively. Assuming that the number of layers of GNN is L, the time and space complexity of GNN are both $O(L * (E + N))$.

The attribute extractor extracts all molecular attributes in a data set at one time, which can be generated directly from pre-generated attributes or directly through the attribute extractor. If it is directly generated through the attribute extractor, the time and space complexity are $O(D * F * T)$ and $O(D * F)$ respectively, where T represents the time required by rdkit to extract a molecular fingerprint, D is the size of the data set and F is the dimension of the attributes.

The dimensions of grpah embedding and attributes are both 100, GNN has 5 layers, and M is a constant, so the time and space complexity of APN is $O(N + E)$, where N is the number of nodes and E is the number of edges. $O(N + E)$ means that the space and time consumption of APN is a linear function of the number of nodes and the number of edges, which is usually the ideal situation for processing graph structures, demonstrating that APN is a very efficient method.

Appendix G. Details of Dataset

The details of tasks in Tox21, SIDER and MUV datasets are as follows: 1) Tox21 contains 12 assays each measuring the human toxicity property of a biological target, including 'NR-AR', 'NR-AR-LBD', 'NR-AhR', 'NR-Aromatase', 'NR-ER', 'NR-ER-LBD', 'NR-PPAR-gamma', 'SR-ARE', 'SR-ATAD5', 'SR-HSE', 'SR-MMP', 'SR-p53'. 2) SIDER records the side effects of compounds, which splits 5868 side effects into 27 categories, including 'Hepatobiliary disorders', 'Metabolism and nutrition disorders', 'Product issues', 'Eye disorders', 'Investigations', 'Musculoskeletal and connective tissue disorders', 'Gastrointestinal disorders', 'Social circumstances', 'Immune system disorders', 'Reproductive system and breast disorders', 'Neoplasms benign, malignant and unspecified (incl cysts and polyps)', 'General disorders and administration site conditions', 'Endocrine disorders', 'Surgical and medical procedures', 'Vascular disorders', 'Blood and lymphatic system disorders', 'Skin and subcutaneous tissue disorders', 'Congenital, familial and genetic disorders', 'Infections and infestations', 'Respiratory, thoracic and mediastinal disorders',

’Psychiatric disorders’, ’Renal and urinary disorders’, ’Pregnancy, puerperium and perinatal conditions’, ’Ear and labyrinth disorders’, ’Cardiac disorders’, ’Nervous system disorders’, ’Injury, poisoning and procedural complications’. 3) MUV is a challenging virtual screening dataset, containing 93127 compounds in 17 assays, such as ’MUV-466’, ’MUV-548’, ’MUV-600’, ’MUV-644’, ’MUV-652’, ’MUV-689’, ’MUV-692’, ’MUV-712’, ’MUV-713’, ’MUV-733’, ’MUV-737’, ’MUV-810’, ’MUV-832’, ’MUV-846’, ’MUV-852’, ’MUV-858’, ’MUV-859’.
